# A discrete‐event simulation model for analysing and improving operations in a blood donation centre

**DOI:** 10.1111/vox.13111

**Published:** 2021-05-06

**Authors:** Martina Doneda, Semih Yalçındağ, Inês Marques, Ettore Lanzarone

**Affiliations:** ^1^ Institute for Applied Mathematics and Information Technology (IMATI) National Research Council of Italy (CNR) Milan Italy; ^2^ Industrial Engineering Department Yeditepe University Istanbul Turkey; ^3^ Center for Management Studies Instituto Superior Técnico University of Lisbon Lisbon Portugal; ^4^ Department of Management, Information and Production Engineering University of Bergamo Dalmine Italy

**Keywords:** blood supply chain, blood collection centre, donor flow, decision support system, discrete event simulation

## Abstract

**Background and objectives:**

Healthcare systems require effective and efficient blood donation supply chains to provide an adequate amount of whole blood and blood components to hospitals and transfusion centres. However, some crucial steps of the chain, for example blood collection, are not adequately studied in the literature. This work analyses the operations in a blood collection centre with the twofold aim of analysing different configurations and evaluating the effectiveness and feasibility of schedules defined at higher planning levels.

**Materials and methods:**

The analyses are performed through a discrete event simulation (DES) model that describes a customizable collection centre. Moreover, a feedback loop from the DES to the higher planning level allows to adjust scheduling decisions if they determine criticalities or infeasibilities at the operational level.

**Results:**

Numerical tests have been conducted considering a real Italian provider. An experimental plan has been designed to compare different configurations for the blood collection centre and evaluate the best ones in terms of cost and service quality for the three main actors involved (donors, workers and managers). The best configurations have been also used to test the feedback loop.

**Conclusions:**

Results confirm the appropriateness of the proposed DES model, which can be considered a useful decision support tool for dimensioning and managing a blood collection centre, either as a standalone tool or in conjunction with a scheduler.

## Introduction

Transfusion medicine is arguably one of the most industrial‐like specialities in modern medical science. Although some studies are ongoing, the state of the art for artificial substitutes of human blood is far from satisfactory, meaning that healthy donors are the only suppliers [1, 2]. Moreover, blood components have a limited shelf life (up to 42 days for red cells, 5 or 7 days for platelets depending on the regulatory environment, and 2 years for plasma), which prevents strategic long‐term storage [3, 4].

Blood is provided to healthcare systems through the so‐called Blood Donation Supply Chain (BDSC), whose management includes both strategic and operational decisions. Following the descriptions provided by Sundaram and Santhanam [5] and Osorio et al. [6], the BDSC can be divided into four stages: (i) collection (including donor registration, donation and blood screening); (ii) transportation; (iii) storage; (iv) utilization (including demand prediction, supply management and distribution).

In particular, collection is one of the most important stages in making the BDSC work properly. In fact, collecting an adequate amount of blood is a key requirement, as an unbalanced supply of units could trigger alternating periods of blood shortage and wastage. Therefore, its proper management is essential to improve all downstream steps, including patient care. Moreover, its management is particularly critical because blood collection merges the characteristics of a production system, where both demand and production are stochastic, and those of a service provider regarding the collection from donors [7, 8]. However, some stages of the BDSC are not adequately addressed in the literature yet. For example, while storage is extensively studied, collection is only marginally considered despite its relevance [9].

Given the increasing global demand for blood and the need to comply with budgetary and legal requirements, there is a growing need to reengineer the BDSC and in particular the management of collection centres by implementing efficient evidence‐based policies. Moreover, as these centres are typically managed by non‐technicians, these policies should be implemented using highly intuitive tools [10].

In this context, our paper focuses on the operational management of blood collection centres with a double motivation. On the one hand, we analyse different centre configurations to identify the best ones in terms of cost and service quality, considering the perspectives of the three main actors involved (donors, workers and managers). On the other hand, we assess the interactions of the operational level with higher planning levels and, in particular, we evaluate the operational effectiveness and feasibility of the schedules deriving from donor appointments defined at the tactical level.

Quantitative analyses are performed through a discrete event simulation (DES) model that describes a general and customizable blood collection centre. The DES architecture was chosen over other methodologies because of its inherent flexibility, the reduced number of assumptions required to mimic reality, and its ability to determine the best configuration among a series of alternatives under uncertainty, by exploiting what‐if analysis. Even if this may lead only to nearly‐optimal policies, those are sufficient in most cases where the optimal solution is indeterminable or impractical: for instance, other methods such as Integer Linear Programming require high computation power and more restrictive assumptions to provide a solution. Moreover, DES gives an intuitive representation of the flows, which can be readily understood by non‐technical personnel without a strong operations research background, as often is the staff in blood collection centres. Finally, it has a short learning curve and allows users to easily verify the effectiveness and feasibility of decisions made at higher levels, enabling optimization‐simulation frameworks in case these decisions need to be revisited.

The structure of the DES model is formalized with Business Process Model and Notation (BPMN) standards [11], while the inputs are provided by a donor appointment schedule. To validate the DES model and demonstrate its practical applicability, numerical experiments have been conducted considering the real case of the Milan branch of the main Italian blood provider, the *Associazione Volontari Italiani Sangue* (AVIS), hereinafter referred to as AVIS Milan. For such a case, the input schedule has been provided by the Blood Donor Appointment Scheduling (BDAS) tool presented in Baş Güre et al. [12] and Yalçındağ et al. [13].

In the literature, one of the earliest works dealing with BDSC from a non‐clinical point of view was proposed by Millard [14], who suggested applying industrial inventory models to blood collection. However, BDSC only began to receive broader attention a decade later, as reported in several literature reviews [9, 15, 16]. Although BDSC has been extensively studied in the operations research literature, the different steps of the BDSC have not received the same attention. For example, as mentioned above, storage is the most addressed step, and collection the least one [9].

Several techniques can be applied to study blood collection, for example evaluation of best practices, linear and integer programming, queuing models and Markov decision processes [17, 18, 19, 20, 21, 22, 23, 24]; however, simulation‐based works outnumbered those with any other solution method over the years. Indeed, simulation is a rather effective approach to address BDSC management problems, according to Beliën and Forcé [15], because of the complexity of the system. It usually leads to the identification of near‐optimal policies, which may be sufficient in cases where the optimal solution is indeterminable or impractical. In particular, DES has been highlighted as the most effective and practical approach for many aspects of BDSC management.

Finally, it is worth mentioning that DES has been successfully applied to the optimization of operations in other healthcare services, for example in operating theatres [25], in specific home care services [26] and in resource allocation for screening [27].

In the following, we focus on simulation techniques applied to the management of a blood collection centre to highlight the differences and underline the contributions of our work.

Jennings [28] used simulation to evaluate the impact of different policies on stock levels, shortages and the expiration of blood units. Pratt and Grindon [29] developed a computer simulation model to study flow and queuing problems arising in blood collection, considering various scheduling strategies. Sirelson and Brodheim [30] built a simulation model to evaluate the performance of a platelet inventory system in terms of out‐of‐stock and expired units. Brennan et al. [31] studied service and productivity problems for American Red Cross blood collection using a general purpose simulation software, with the goal of understanding which donor arrival patterns make the system more efficient under different configurations, staff allocations and work rules. Michaels et al. [32] studied the impact of several planning strategies to schedule the arrival of donors at a corporate blood collection. More recently, De Angelis et al. [33] integrated a simulation model with a neural network to find an optimal resource configuration for a transfusion centre in Rome, modelling the operations as a set of consequential servers with a given nominal capacity. Rytilä and Spens [34] simulated the Finnish transfusion system, while Katsaliaki and Brailsford [35] analysed a British hospital supplied by a regional blood centre. Alfonso et al. [36] applied Petri net models and quantitative simulation to the case of a blood collection centre in France, defining performance indicators to evaluate human resource planning and donor arrival patterns both for booked apheresis donors and walk‐in whole blood donors, in mobile and in fixed collection settings alike. Blake and Shimla [37] used a flow shop model to create a linear model that determines the most efficient staffing configuration in a Canadian blood centre. Finally, Moons et al. [38] exploited a software designed for industrial simulation to determine optimal staffing and resource planning.

According to Pirabán et al. [16], no work dealing with the simulation of a blood collection centre implemented an optimized appointment schedule so far. In fact, the available models are intended to identify the best input management policies given a set of organizational constraints, while in our work we focus on merging simulation with the output of an optimized scheduler. Some of the listed works also included a visual representation of the simulator, to make its functioning more intuitive for the managers of blood donation centres, who usually are medical (non‐technical) personnel [10]. Some also considered multiple stakeholders’ perspectives to evaluate their results. Therefore, to make its functioning more intuitive, our tool also includes a visual representation of the simulated environment.

Table 1 summarizes the main characteristics of the studies dealing with simulation for blood collection and compares them with our work. None of the other published papers included all the characteristics that are considered together in our work, and in particular the integration with an optimized schedule. Among the available works, the one proposed by Alfonso et al. [36] is the most similar to ours, but this also neglects the just‐mentioned feature.

**Table 1:** Characteristics considered in the available works dealing with a simulator for blood collection and in this work: booked donors (BD); unbooked donors (UD); fixed setting (FS); mobile setting (MS); visual representation (V); assessment of three stakeholders’ perspectives (P); interaction with optimized schedule (OS)

	BD	UD	FS	MS	V	P	OS
Pratt and Grindon [29]		X		X			
Brennan et al. [31]		X		X			
Michaels et al. [32]	X	X		X			
De Angelis et al. [33]		X	X		X		
Alfonso et al. [36]	X	X	X	X	X	X	
Blake and Shimla [37]	X	X	X	X		X	
Moons et al. [38]		X	X		X		
This work	X	X	X		X	X	X

The remainder of this paper is structured as follows. Section 2 describes the proposed DES model, its implementation, validation and application to the AVIS Milan case. Section 3 presents the results for both the analysis of alternative layouts and the feedback to the appointment scheduler. Finally, Section 4 and 5 discuss the results and provides the conclusion to the work.

## Materials and methods

The operations at the blood centre are based on the observations made in AVIS Milan and integrated with those reported in the literature, mainly in Alfonso et al. [36]. The resulting description is general and customizable enough to describe several blood collection centres.

### Process description

The whole blood donation process for a donor generally consists of four main steps:

1. All donors who arrive at the donation centre show up at the reception, where an employee records their information. Donors are also given a pre‐donation anamnestic questionnaire to fill out.
2. Donors enter a consultation room with a physician. Relevant vital signs are measured, including blood pressure, heart rate and haemoglobin concentration. After all data have been collected, the physician determines whether each donor can donate that day or whether they must be deferred temporarily or permanently. If not deferred, donors can proceed with the donation.
3. Donors are assigned to a donation bed, where all necessary devices and consumables have been prepared. The skin of the donor's inner elbow is disinfected, a vein of the arm is punctured and the phlebotomy begins. The first millilitres of blood are collected in test tubes, which will be sent for screening. Then, the withdrawn blood is directed to the donation bag. After the target extraction level (∼450 ml) is reached, the needle is removed and donors are left on the bed for a few additional minutes. This prevents vagal fainting that may occur when the donor suddenly switches from lying to standing [39].
4. Finally, donors are directed to a canteen area to get refreshed, and above all for post‐donation supervision, to detect possible negative outcomes before they leave the centre.

All works report that the blood collection process begins with donor registration and ends with the donor being offered a light meal after phlebotomy in a canteen or snack room. However, other activities such as questionnaire filling, haemoglobin testing, vital signs check and clinical counselling are taken into account differently in each work. For example, haemoglobin can also be measured before filling out the questionnaire or in the donation room just before the donation.

The arrival of donors can occur in different ways and according to different rules. In our DES model, input arrivals are given by an appointment schedule and in particular by the BDAS scheduler of Baş Güre et al. [12], whose aim is to balance the production of the different blood types (combination of group and Rhesus factor) between days while penalizing overtime and periodic accumulation of donors (queues). This scheduler divides each day into k periods (e.g. ‘early morning’, ‘late morning’ and ‘early afternoon’) considering already booked appointments and the expected number of walk‐in (unbooked) donors for each period to pre‐allocate group‐specific donation slots with the aim of balancing blood type production. Then, when donors make a reservation, they fill one of these slots. The system is dimensioned on the basis of the overall physician capacity for each period and standard time for a medical consultation and is capable of penalizing donor accumulation using period‐specific weights. The choice of physician time as the scarce resource derives from the characteristic of the system under analysis. Indeed, different from other health facilities where beds represent the bottleneck of the system, the cost for adding a physical bed, if there is room, is not so relevant for the considered system. Here the highest costs are associated with disposables, tests performed on donated units, and staff; while the former two items are fixed, given the number of units, staff represents the most relevant cost item on which a proper management of the blood collection centre may have an impact. Finally, if the number of booked donors for a given blood type is high, the scheduler dynamically reacts by increasing the number of new slots to allocate to that given blood group rather than simply allowing overbooking. The interested reader is referred to Baş Güre et al. [12] for more details.

The following assumptions have been made to formalize the design of the DES model. Only donations at a fixed‐site blood collection centre are included, while mobile settings are excluded (e.g. blood collection vehicles sent by the centre for collection in schools, companies and at events). Only whole blood donations are considered, and they are all assumed to be successful, because the actual usability of a donation does not have an impact on the system in terms of resource occupation. A given percentage of booked donors is expected to not show up at the assigned appointment. Moreover, a given percentage of donors is estimated to be deferred from the donation after being visited; these donors leave the centre without going through the next steps and will return from a date set by the physician. Apart from this, donors do not voluntarily leave the process once they enter, contrary to what was assumed by Alfonso et al. [36]; instead, we model queues without a maximum waiting time and use the time spent in the queue as a performance metric.

The DES model refers to voluntary centres in Western countries; however, international standards contribute to unify the key steps, and the World Health Organization is actively promoting homogeneity among all transfusion systems [2, 40].

The DES includes the following configurations to cope with the alternative layouts for the activities in steps 1 and 2, to guarantee generality and flexibility to the description:

- Questionnaire filling: it is combined with waiting and physician’s consultation times.
- Haemoglobin measurement: it can be performed either in conjunction with the consultation or separately, before or after it.
- Vital signs measurement: it is included in the consultation.
- Consultation: it is the last step before phlebotomy, performed in a physician’s office or equivalent setting. It must be remarked that, in some contexts, consultation can be performed by non‐physician personnel. However, this is not a decision that can be taken individually by a blood centre, as it intertwines with local legislations, and, moreover, it does not impact on queuing or staff deployment.

### Conceptual modelling with BPMN notation

A BPMN scheme of the system has been constructed to serve as the basis for the DES model. The BPMN notation allows to present the actions taken by the different actors involved in the process separately and to easily show how they interact and at which stage of the process [11].

**Fig. 1:**
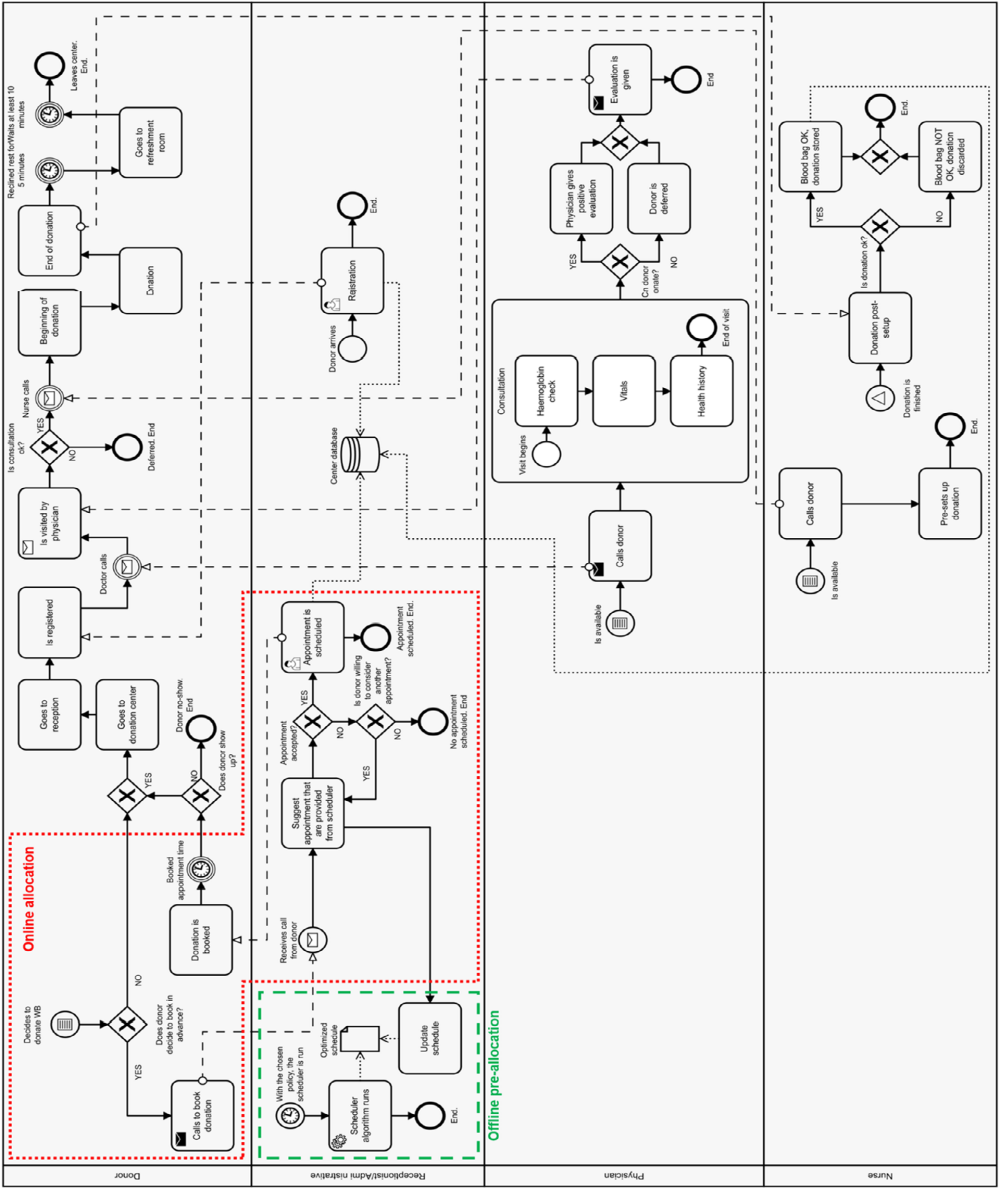
BPMN model of the operations carried out at the blood collection centre.

The BPMN diagram is shown in Fig. 1. Each horizontal section is devoted to the actions taken by an actor: (i) donors, (ii) receptionists and administrative staff, (iii) physicians and (iv) nurses. The two blocks within coloured lines refer to the activities carried out at a higher level by the BDAS [12], included here as they characterize the system input. In particular, the green dashed line and the red dotted line identify the offline pre‐allocation and the online allocation phases, respectively. All logical blocks, included in the DES model, represent the actual blood donation process according to the 4 steps presented in Section 2. Summing up, during the reception phase the donors arrive at the centre and interact with an administrative figure (receptionist or employee), who registers their arrival. The medical consultations are done by physicians, who decide whether each donor can donate that day or whether they should be deferred. During the phlebotomy, donors are assisted by a nurse, who takes care of the pre‐setup and post‐setup of the collection equipment. Finally, donors get a refreshment before leaving the facility.

### Model implementation

**Fig. 2:**
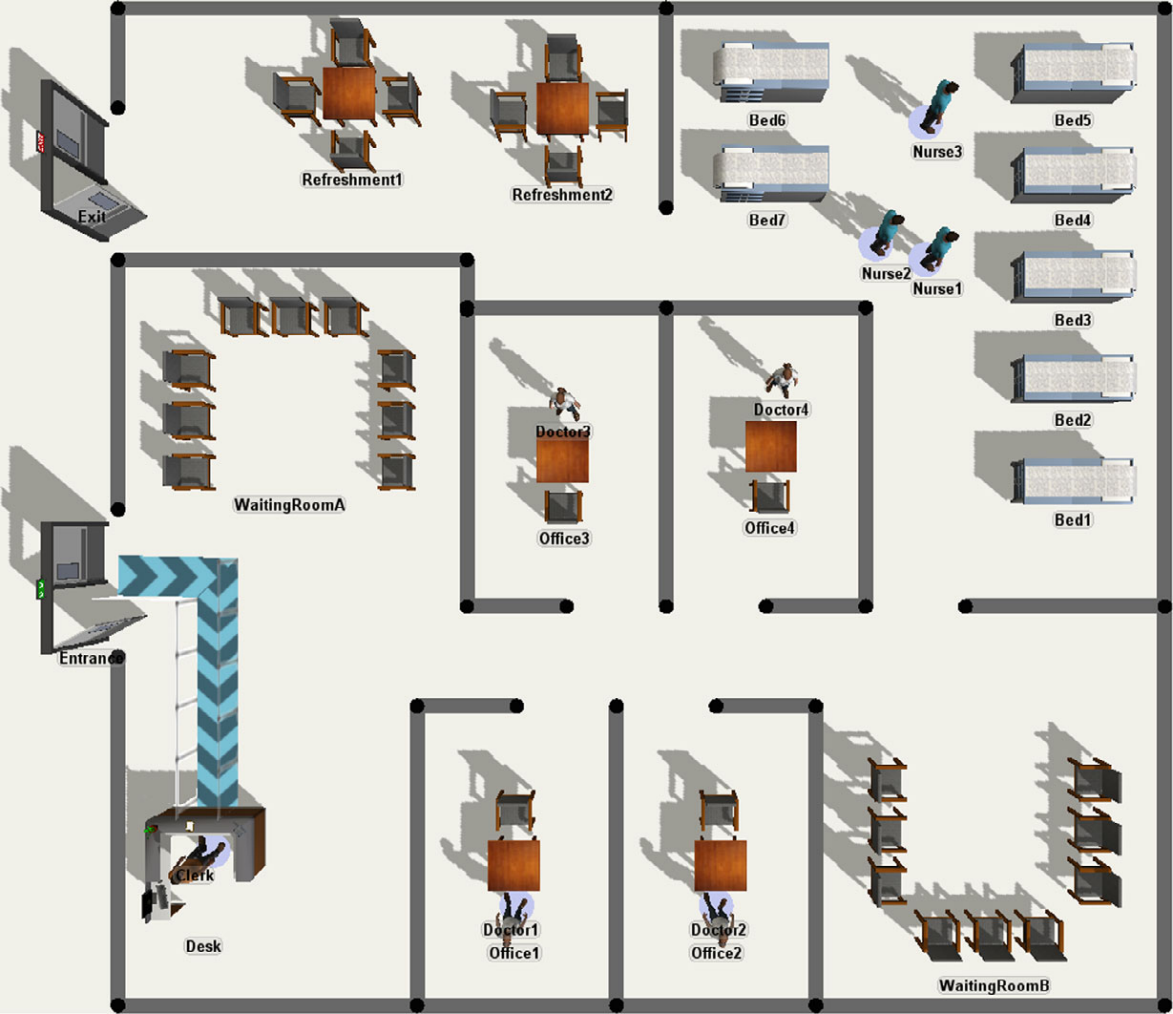
Bird’s eye view of the 3D environment of the simulator.

The DES model has been implemented in FlexSim (FlexSim Software Products Inc., Orem, UT, USA), in a parametric form so that the parameters of the distributions can be easily adjusted or tailored. This implementation also provides a real‐time 3D visual counterpart of the simulated environment, shown in Fig. 2.

The arrivals of both booked and unbooked donors at the centre are taken for each period from the BDAS pre‐allocation solution. As for booked donors, their arrival times are generated using a uniform probability distribution within their respective period. Since the scheduler assigns donors to periods, rather than giving them a specific appointment, the uniform distribution provides a satisfactory and unbiased proxy for the behaviour of booked donors, reflecting the situation of AVIS Milan in which no particular donor arrival pattern has been observed. By directly considering the BDAS pre‐allocation as input rather than the actual reservations of donors who call to make a reservation (see Baş Güre et al. [12]), not all pre‐allocated slots are expected to be filled and converted into booked donors’ slots. Therefore, each pre‐allocated slot is considered to become booked following a Bernoulli distribution with probability $p_{\mathrm{fill}}$. In addition, some booked donors may not show up for the donation with respect to the nominal arrivals. To model this, each booked donor is considered not to show up following a Bernoulli distribution with probability $p_{\mathrm{ns}}$. Finally, unbooked donors are generated in quantities equal to the expected value considered in the BDAS pre‐allocation solution, within their respective period, using a uniform probability distribution for the arrival time. This also reflects the situation of AVIS Milan where these donors tend to show up randomly.

The activities are organized and modelled as detailed below.

Each donor goes immediately to the registration desk upon arrival. Registration is set up to run differently depending on whether the donor is booked or not. In particular, the registration time is longer for unbooked donors, due to the fact that their data must be entered into the database by the clerk who serves them, and this process takes additional time.

After being successfully registered, donors must see a physician for consultation. Each physician is located in a separate office. If there is a free physician, donors can access the office directly; otherwise, they must stay in a waiting area until their turn comes. Different logics for queue management have been considered in the DES model; in the baseline scenario, the queue is served using a *First* *In First Out* (FIFO) logic. During the waiting time, donors fill out a questionnaire they received during registration; therefore, the time for compilation is not modelled separately.

When a physician is available, donors go to his/her office and undergo a consultation. One aspect of the assessment is haemoglobin measurement, which as mentioned can be carried out together with the consultation or not. It is considered jointly in the baseline scenario, and separately in the variants. The two process times (consultation and haemoglobin measurement) are modelled separately; in the baseline scenario, we consider their sum.

The outcome of the clinical assessment for donation eligibility depends on whether donors are booked or not. In fact, unbooked donors are assumed to have a rejection rate at least five times higher than the booked ones, who receive initial feedback during the reservation and are more familiar with the reasons why one should self‐abstain from donating. Separately for the two types of donors, rejections are modelled using Bernoulli distributions with different parameter values.

Afterwards, accepted donors enter the donation room. The first available nurse accompanies them to the first available bed according to a FIFO policy in all scenarios. Then, the pre‐setup of the machine is performed by the nurse, including the puncture of the donor’s vein.

During phlebotomy, the nurse is able to attend other activities that need to be performed, including taking care of other donors. After the donation, when the planned amount of blood has been withdrawn, a nurse is again engaged to disconnect the donor from the bag and for the post‐setup activities.

After successfully completing the donation, donors are asked to rest on the bed to avoid vasovagal fainting related to the orthostatic reflex. Then, they can leave the donation room and move to a canteen area, where they have a refreshment before leaving the centre. This last step is not fundamental to the workflow of the donation centre, especially because it is managed separately and usually organized as a self‐service area. However, the time spent in the canteen is an integral part of the psychological time that donors spend inside the centre. In this light, the refreshment phase is included in the calculation of the total donors’ cycle time.

### Experimental setting

A first set of experiments was conducted to validate the DES model. Then, an experimental plan was designed to compare different configurations and evaluate the best ones in terms of cost and service quality for the three main stakeholders involved (donors, workers and managers). Finally, a feedback loop from the DES to the higher scheduling level was implemented and evaluated, to adjust scheduling decisions if they determined criticalities or infeasibilities at the operational level. More specifically, the feedback consisted in the re‐calibration of some parameters used by scheduler, based on the outcomes obtained from the DES model.

All experiments have been tailored to the AVIS Milan case, in terms donor flow and number of resources involved.

In the following, after reporting the adopted parameters and the Key Performance Indicators (KPIs) designed to evaluate the performance of the collection centre in Sections 2.4.1 and 2.4.2, respectively, we present the validation of the DES model in Section 2.4.3 and the tested alternative layouts in Section 2.4.4.

### Simulation parameters

Already booked donors, pre‐reserved slots and expected walk‐in donors were taken from the first day solution of the BDAS scheduler was taken in Baş Güre et al. [12]. In particular, we randomly extracted a solution generated after the ramp‐up period from the scheduling experiments reported in that work. Moreover, as similar amounts of donors were observed in the BDAS between days, the solution is representative of the considered collection centre.

The distributions of the different process times used are from the available literature. Since AVIS Milan could not provide us all the data required to build the whole simulation model, we referred to the literature as a proxy. The adopted distributions were evaluated against qualitative observations reported by AVIS Milan, and it was observed that the two sets were comparable. On this basis, process times were defined as follows:

- Registration for booked donors: normal distribution with mean value $\mu_{\mathrm{ru}}=1\cdot 77$ and standard deviation $\sigma_{\mathrm{rb}}=0\cdot 612$ (Alfonso et al. [36]).
- Registration for unbooked donors: log‐normal distribution with mean value $\mu_{\mathrm{rb}}=2\cdot 4$ and standard deviation $\sigma_{\mathrm{ru}}=0\cdot 82$ (Brennan et al. [31]).
- Consultation: triangular distribution with minimum value $a_{c}=16\cdot 5,$ maximum value $b_{c}=20$ and modal value $c_{c}=18\cdot 33$ (shape from Alfonso et al. [36], with parameters modified to fit the AVIS Milan case).
- Haemoglobin testing: uniform distribution between minimum value $m_{h}=1\cdot 34$ and maximum value $M_{h}=2$ (Alfonso et al. [36]).
- Pre‐setup of collection equipment: normal distribution with mean value $\mu_{s1}=1\cdot 6$ and standard deviation $\sigma_{s1}=0\cdot 284$ (Alfonso et al. [36]).
- Phlebotomy: Weibull distribution with scale $\lambda_{p}=4\cdot 23$, shape $k_{p}=1\cdot 82$ and location $\gamma_{p}=9\cdot 5$ (Alfonso et al. [36]).
- Post‐setup of collection equipment: uniform distribution between minimum value $m_{s2}=1$ and maximum value $M_{s2}=2$ (Alfonso et al. [36]).
- Resting: triangular distribution with minimum value $a_{l}=4$, maximum value $b_{l}=10$ and modal value $c_{l}=5$ (Tomasulo et al. [39]).
- Post‐donation refreshment: log‐normal distribution with mean value $\mu_{r}=14\cdot 22$ and standard deviation $\sigma_{r}=7\cdot 11$ (Alfonso et al. [36]).

Note that parameters $a_{c}$, $b_{c}$ $c_{c}$, $m_{h}$ and $M_{h}$ related to the consultation and haemoglobin distributions are calculated such that the expected value of the sum of these two times is equal to the standard consultation time used in the BDAS pre‐allocation.

The k=3 periods used in the BDAS solution were translated into 3 blocks of two hours: 7:30 a.m. to 9:30 a.m.; 9:30 a.m. to 11:30 a.m.; 11:30 a.m. to 1:30 p.m.. However, the simulation did not stop at the end of the third period to measure the time the last donor leaves the system after completing the donation process, even if it falls after the centre closes. In this way, it was possible to assess whether queuing causes some donors to remain in the system beyond opening hours.

Moreover, the following parameters were taken for the Bernoulli densities: no‐show rate of already booked donors $p_{\mathrm{ns}}=0\cdot 05$; filling rate of pre‐allocated slots $p_{\mathrm{fill}}=0\cdot 9$; deferral rate at the consultation for booked donors equal to $5\cdot 03\times 10^{‐3}$; deferral rate at the consultation for unbooked donors equal to $4\cdot 523\times 10^{‐2}$; deferral rate at the haemoglobin testing equal to $5\times 10^{‐3}$ for both booked and unbooked donors. Deferral rates were chosen to give a total acceptance rate of 99% and 95% for booked and unbooked donors, respectively, as provided by AVIS Milan. These high acceptance rates are associated with the fact that booked donors are usually pre‐screened during reservation and with the fact that also unbooked donors are regular donors aware of the acceptance criteria for donating.

Finally, the other parameters used in the DES model are in agreement with the BDAS solution considered: collective nominal physician capacity equal to 450 min for all periods; mean consultation time equal to 20 min.

### Key performance indicators

Several KPIs have been identified taking into account the point of view of the three main stakeholders involved: donors, staff and managers of the centre, each one with their own prerogatives. Targets regarding KPIs were discussed with AVIS Milan, but different centres with different needs may personalize their own set of KPIs to reflect the individual needs of the centre.

Two indicators have been identified for donors: total time spent in the system (cycle time, also used in Section 2.4.3 for validation), and time spent in queue. They have also been differentiated between booked and unbooked donors. In particular, cycle time (encompassing all donors) has been our main metric of interest.

Utilization statistics have been considered for resources: three types of personnel (physicians, nurses and clerks) and beds. As for physicians, who are considered the scarce resource, the aim is to limit their utilization, while for the other resources the target is to increase it. This is in agreement with the DBAS scheduler we used.

The main indicator from the management point of view is cost‐efficiency, where efficiency is evaluated in terms of donors’ cycle times. Moreover, two other indicators have been considered in each period k to evaluate the periodic accumulation of donors in the bottleneck of the system (the queue before consultation): the used physician capacity, and the exit time from consultation of the last donor of the period.

A summary of all KPIs is provided in Table 3.

A non‐monetary cost unit (CU) has been associated with all variable resources, ignoring all fixed costs that do not vary with the layout (e.g. structures and physicians, whose number is constant as the layout changes). In particular, the following CUs have been considered, in agreement with Alfonso et al. [36]: clerk salary equal to 5 CUs/workday; nurse salary equal to 10 CUs/workday; the cost of a bed equal to 1 CU/workday. In the AVIS Milan case, 1 CU corresponds to about 20 euros.

### Model validation

The DES model was verified and validated in two stages.

First, a face validation and a verification for reasonableness analysis were performed. Face validation was carried out iteratively, comparing model results with feedback from subject‐matter experts [41] from AVIS Milan, and adjusting parameters accordingly (the above presented parameters are already those set after validation). As for the verification, the inputs were varied and the adequacy of the system response was verified, for example we checked that queue length and delays increased/decreased as donor arrival rates increased/decreased. System sensitivity to different probability distributions was also checked and found to be low.

Second, and more importantly, a statistical validation of model performance was performed by considering n=5 replications of the baseline DES solution, modelling the current configuration of the centre, and focusing on the main metric of interest, that is the donor cycle time (referred here as $T_{D}$). In fact, it is a comprehensive metric, able to define the throughput of the collection centre. AVIS Milan reported that the true value of $T_{D}$ for their centre is μ=65 minutes. First, we tested the 5 replications for normality. The sample mean for donor cycle time (${\underline{X}}_{T_{D}}$) was 71·798, and its sample standard deviation ($S_{T_{D}}$) was 7·634. Then, we conducted a 2‐tailed *t*‐test with significance level α=0·05, testing the null hypothesis $H_{0}$ that $E\left(X_{T_{D}}\right)=\mu$. The test failed to reject $H_{0}$, meaning that the DES adequately models the system. However, failing to reject $H_{0}$ is a weak conclusion, unless the power of the test is high. Therefore, we calculated the sample critical difference $\hat{\delta }$ for power analysis:

$$\hat{\delta }=\left|\frac{{\underline{X}}_{T_{D}}‐\mu }{S_{T_{D}}}\right|=\left|\frac{71\cdot 798‐65}{7\cdot 634}\right|=0\cdot 890$$

Referencing an operating characteristic curve (OC curve), the probability of a type‐II error $\beta \left(\hat{\delta }\right)$ was around 70% with n=5, an unacceptably high value. According to the OC curve, to obtain $\beta \left(\hat{\delta }\right)$ less than 0·05, n=30 samples were required. Therefore, n=30 replications were used in all experiments to guarantee the significance of results.

### Alternative layouts

The following parameters and policies were tested in the experiments:

- Queuing policy: all donors are treated equally (FIFO setting of the baseline scenario) or booked donors are prioritized with respect to unbooked ones. In this case, booked donors are always called before any other unbooked donor already in the queue for the consultation.
- Haemoglobin testing: it is placed within, before or after consultation. In the latter two cases, a designated area for testing must be set up, with personnel dedicated to carrying out the test. The additional unit of personnel is assumed to be a nurse.
- Planned capacity of physicians in each period k: since the considered capacity $c_{\mathrm{tk}}$ of 450 min corresponds to 3·75 physicians over a two‐hour period, three physicians working for the whole period and one working for an hour and a half are required. This hour and a half can be placed either at the beginning or at the end of the period, resulting in two possible configurations.
- Capacity of other resources not included in the BDAS: the number of clerks was set at 1 or 2, the number of nurses at 2 or 3, and the number of beds at 5, 6 or 7, varying on the baseline of the AVIS Milan case. We assumed that, apart from the physicians, all personnel are present in the blood collection centre throughout the day from the beginning of the first period to the closure.

These are the parameters that can have a significant impact on system performance, both looking at the DES model and from considerations made by the AVIS Milan staff, who suggested the above‐mentioned factors and levels.

The combination of these factors produced a total of 144 alternative layouts, as summarized in Table 2. Each layout was tested n=30 times, for a total of 4,320 runs. Repetitions were performed assuming common random numbers as the layouts varied, by repeating the simulations with exactly the same streams of uniforms random numbers. The name of each layout is uniquely identified by a string that includes the name of the alternatives as in Table 2. For example, the layout ‘FIFO‐1‐W‐01’ uses the FIFO queuing policy, has the fourth physician working at the beginning of each period and the haemoglobin test performed during the consultation, and includes 2 nurses, 1 clerk and 5 beds.

**Table 2:** Alternative parameters and policies tested in the experiments

Parameter	Alternatives			Name
Queuing policy	FIFO			FIFO
	Priority to booked donors			PRTY
Physician timetable	At the beginning			1
	At the end			2
Haemoglobin testing	Within consultation			W
	After consultation			A
	Before consultation			B
Number of resources	*Nurses*	*Clerks*	*Beds*	
	2	1	5	01
	2	1	6	02
	2	1	7	03
	2	2	5	04
	2	2	6	05
	2	2	7	06
	3	1	5	07
	3	1	6	08
	3	1	7	09
	3	2	5	10
	3	2	6	11
	3	2	7	12

The last column reports the name of the alternative, used to compose the name of the scenario.

## Results

This Section presents the results from both the analysis of alternative layouts and the feedback to the BDAS.

### Analysis of alternative layouts

We performed a multivariate anova for the KPIs, including the six factors reported in Table 3 (queuing policy, physician timetable, haemoglobin testing, number of nurses, number of clerks and number of beds) with their respective levels, and all possible two‐factor interactions. The cost‐efficiency KPI was excluded from this first part of the analysis, as it is a function of cycle time and the cost associated with the number of resources involved. Calculations were made using MATLAB (version R2020a, Mathworks, Natick, MA, USA).

**Table 3:** KPIs analysed in the DES solutions

Stakeholder		KPI	Notes
Donors		Cycle time	Stratified for booked/unbooked
		Queue time	Stratified for booked/unbooked
Resources	Nurses	Utilization	Goal is to saturate
	Clerks	Utilization	Goal is to saturate
	Beds	Utilization	Goal is to saturate
	Physicians	Utilization	Goal is to desaturate
Management		Capacity	Physician capacity actually used, per period
		Last donor	Exit time from consultation, per period
		Cost‐efficiency	Efficiency in terms of donors’ cycle times

To properly employ anova, the normality of the KPIs over the repetitions was checked by analysing their respective q‐q plots. Although some outliers were present, the q‐q plots showed that a normal distribution can be assumed for the sampled data, which allowed to correctly use anova for all KPIs. A summary of the statistically significant factors is reported in Table 4, along with the statistically significant two‐factor interactions.

**Table 4:** p‐values from the anova for the significant factors and interactions

			Main effects						Interactions				
			Queueing policy	Physician timetable	Haemoglobin testing	Number of nurses	Number of clerks	Number of beds	Queuing ∙ Haemoglobin	Queuing ∙ N° of beds	Timetable ∙ N° of nurses	Haemoglobin ∙ N° of nurses	N° of nurses ∙ N° of beds
Donors	Cycle time			**0·0002**	**0·0000**		0·0243	**0·0000**					
	Cycle time – booked		**0·0000**	**0·0009**	**0·0000**		*0·0044*	**0·0000**					
	Cycle time – unbooked		**0·0000**	*0·0029*	**0·0000**			0·0196					
	Queue time		**0·0000**	**0·0006**	**0·0000**			**0·0000**	0·0112				
	Queue time – booked		**0·0000**	**0·0006**	**0·0000**			**0·0000**	**0·0000**				
	Queue time – unbooked		**0·0000**		**0·0000**			**0·0000**		**0·0007**			
Resources	Nurses’ utilization			**0·0008**	**0·0000**	**0·0000**	0·0235	**0·0000**			**0·0007**	**0·0000**	**0·0000**
	Clerks’ utilization						**0·0000**						
	Beds’ utilization							**0·0000**					
	Physicians’ utilization				**0·0000**								
Management	Capacity	k = 1			**0·0000**								
		k = 2			**0·0000**								
		k = 3			**0·0000**								
	Last donor	k = 1	**0·0000**		**0·0000**								
		k = 2		**0·0006**	**0·0000**								
		k = 3		**0·0000**	**0·0000**								

The different significance levels are highlighted as follows: 0·001 in bold, 0·01 in *italics* and 0·05 without any formatting.

Regarding donors, queuing policy was found to have a very strong impact (p‐value = 0·0000). The prioritization policy regularized booked donors’ cycle times, reducing both their dispersion and mean value (on average, in the order of a minute). However, at the same time, the prioritization policy created a population of unbooked donors for which the cycle time increased to over 2 h. This is undesirable for unbooked donors because a bad service experience is likely to push potential periodical donors out of the system, resulting in a net loss. If strictly judged from the operational point of view, the prioritization policy in this form should be avoided, considering the small difference in booked donors’ average cycle time between the alternatives. However, on the one hand, giving equal priority to both types of donors may undermine the scheduling system itself. On the other hand, AVIS Milan observed that too long waiting times discourage new and occasional donors to become regular. Therefore, the decision maker should find a balance between these two aspects. We simulated both scenarios to quantify the impact of prioritizing booked donors.

Experiments also showed that having more physician capacity towards the end of the period (configuration ‘2’) significantly helped to disperse queues more quickly (p‐value = 0·0002). In particular, this is true in collection centres where the population of booked donors is prevalent, and no queues before opening hours are observed.

Considering haemoglobin testing, both configurations ‘A’ and ‘B’ helped to provide a better service (p‐value = 0·000). In fact, although it is more expensive to create a new station which also generates a new queue for donors, these configurations free up physicians, who are considered the bottleneck of the system by the upper‐level scheduler. In particular, screening donors before the consultation (configuration ‘B’) allows to defer anaemic donors before they consume valuable screening resources, in addition to freeing up physician time. As confirmed by AVIS, it may be important to carry out the consultation to donors even when their haemoglobin is low and a donation is not possible on that day. In the case of periodic donors, in fact, a consultation may help them take the necessary actions to get well soon and return to donating as soon as possible.

However, regarding resources, even in the best‐case layout, physicians are only saturated at approximately 70% of their capacity, a quantity lower than the utilization of nurses. This is due to the fact that the scheduler is focused on optimizing with respect to physicians’ capacity. However, it is important to note that the saturation only takes into account physician time directly devoted to consultation. Operations such as compiling paperwork, assisting colleagues or other activities that normally occur in a real collection centre (including breaks) make up the remaining 30%. This is true for the other resources as well. Moreover, not reaching saturation values close to 100% reduces burnout risk and improves workplace satisfaction, so this value is not considered low or undesirable.

In this sense, haemoglobin testing is the only factor significantly impacting physicians’ utilization (p‐value = 0·0000). Indeed, as expected, performing the haemoglobin tests separately results in less saturated physicians. Concerning the exit time from the consultation of the last donor, queuing policy has an impact only in the first period k=1 (p‐value = 0·0000), when queues are still forming, while physicians’ timetable configuration ‘2’, which allows queues to disperse better, becomes significant for periods k=2 and k=3 (p‐value = 0·0006 and p‐value = 0·0000, respectively).

Based on the significant factors highlighted in Table 4 and the above discussions, Table 5 summarizes the best combination of significant factors for each KPI.

**Table 5:** Best combination of factors for each KPI, divided by stakeholder, including only the factors with a statistical significance for the KPI and excluding interactions

			Queueing policy	Physician timetable	Haemoglobin testing	Number of nurses	Number of clerks	Number of beds
Donors	Cycle time			2	B or A		2	7 or 6
	Cycle time – booked		PRTY	2	B		2	7 or 6
	Cycle time – unbooked		FIFO	2	B or A			7 or 6
	Queue time		PRTY	2	B or A			5
	Queue time – booked		PRTY	2	B or A			5
	Queue time – unbooked		FIFO		B or A			5
Resources	Nurses’ utilization			2	B or W	2	1	7 or 6
	Clerks’ utilization						1	
	Beds’ utilization							5
	Physicians’ utilization				B			
Management	Capacity	k = 1			B			
		k = 2			B			
		k = 3			B or A			
	Last donor	k = 1	FIFO		A or B			
		k = 2		2	A			
		k = 3		2	A			
	Cost‐efficiency		PRTY	2	B	2	1	6 or 7

**Fig. 3:**
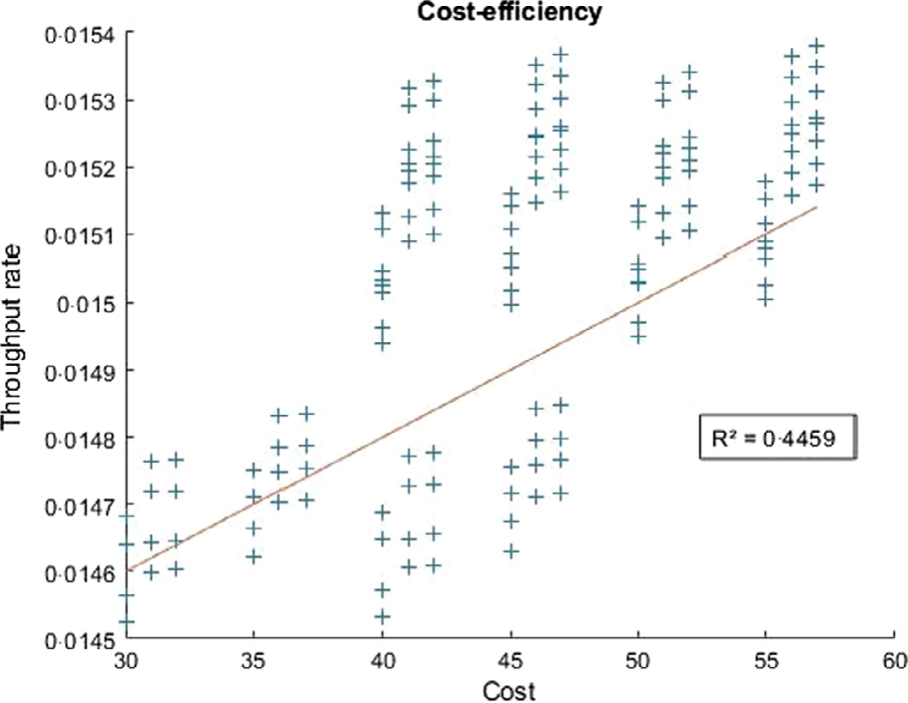
Cost‐efficiency trend: raw data and linear regression trend. [Colour figure can be viewed at wileyonlinelibrary.com]

As mentioned, cost‐efficiency was analysed separately. Figure 3 presents the results of the cost‐efficiency analysis, with costs on the x‐axis and donor throughput rate (the inverse of cycle time) on the y‐axis; moreover, the line represents the linear regression of the data plot. As expected, the system responded with higher throughput rates (lower cycle times) for higher costs, thanks to the greater number of resources invested. However, the R^2^ square of the regression is fairly low, qualifying only into a moderate correlation [42]. For a deeper cost‐efficiency analysis, we used isocost curves, considering the best‐performing configuration at a fixed cost. This allowed us to highlight an interesting pattern, as the best configurations for different isocost levels were similar to each other.

Based on these results, the best‐performing layout was identified for each stakeholder: for donors, *FIFO‐2‐B‐10*; for resources, *FIFO‐2‐B‐03* and for management, *PRTY‐2‐B‐02*.

### Feedback

Some sub‐optimal behaviours in the DES could be traced back to the fact that the BDAS framework was designed to take into account only the midterm objectives of the collection centre, without considering the actual implementation of the plans at the operational level. Therefore, parameters and constraints of the BDAS must dynamically adapt to address the main criticalities that could arise in the daily execution of the activities, that is observed in the DES model outputs.

The first consideration was to revise the consultation time r in the BDAS when considering configurations in which the haemoglobin test is performed separately (configurations ‘A’ and ‘B’). More specifically, the value of the standard consultation time (20 min) should be changed to 18·33 min, that is the mean value of the triangular distribution associated with the consultation only. The second feedback from the DES was that the first period k=1, in which the system is still warming up, resulted undercharged. In opposition, both periods k=2 and k=3 were overcharged, exceeding their respective nominal physician capacities (450 min). To change that, it was possible to intervene in the BDAS by changing the values of the period‐specific penalties associated with the periodic accumulation of donors. We used the same set of weights of the original solution in the inverse order, to penalize the formation of queues more in the third period and less in the first one. All other parameters were left unchanged.

The analysis of the feedback loop was performed for the three best configurations. The new results from the DES with the modified input were then compared with the original ones. To grant a fair comparison, the BDAS was forced to produce an output with the same total number of donors for each day as the original BDAS solution investigated.

Minimum, average and maximum values of the KPIs were compared between pre‐ and post‐feedback run. Results are reported in Table 6.

**Table 6:** Results of feedback on the KPIs in the three configurations analysed

			FIFO‐2‐B‐10			FIFO‐2‐B‐10 (Feedback)			FIFO‐2‐B‐03			FIFO‐2‐B‐03 (Feedback)			PRTY‐2‐B‐02			PRTY‐2‐B‐02 (Feedback)		
			Min	Avg	Max	Min	Avg	Max	Min	Avg	Max	Min	Avg	Max	Min	Avg	Max	Min	Avg	Max
Donors	Cycle time		61·2	66	78·6	62·2	66·3	72·8	61·0	65·4	77·1	61·3	65·7	71·1	60·9	65·3	77·2	61·3	65·5	70·9
	Cycle time – booked		60·8	66	79·1	61·9	66·4	72·5	60·4	65·5	77·3	61·5	65·8	70·6	60·3	64·8	72·1	61·5	65·6	70·6
	Cycle time – unbooked		60·8	66·2	76·4	60·6	66	77·5	59·3	64·7	76·1	59·9	65·2	73·7	59·1	67·8	120·2	59·9	65·0	72·9
	Queue time		4·0	7·3	15·7	4·6	7·3	12·6	4·3	9·0	22·0	5·2	9·1	17·1	1·6	7·0	21·8	1·0	2·7	4·2
	Queue time – booked		4·0	7·3	16·1	4·5	7·4	12·6	4·2	9·1	22·8	5·0	9·3	17·6	1·7	5·8	13·3	1·0	2·5	3·8
	Queue time – unbooked		3·0	7·1	13·8	3·5	6·6	12·5	2·0	8·7	18·8	2·8	7·8	17·0	0·9	12·5	74·2	0·9	2·0	4·9
Resources	Nurses’ utilization		0·62	0·85	1	0·66	0·84	1	0·88	0·96	1·00	0·91	0·97	1·00	0·89	0·96	1·00	0·91	0·97	1·00
	Clerks’ utilization		0·11	0·13	0·14	0·12	0·13	0·14	0·23	0·25	0·27	0·23	0·26	0·28	0·23	0·25	0·27	0·23	0·26	0·28
	Beds’ utilization		0·54	0·62	0·68	0·55	0·62	0·69	0·38	0·44	0·49	0·39	0·45	0·49	0·45	0·51	0·57	0·46	0·52	0·58
Management	Capacity	k = 1	329	368	414	354	399	452	329	368	414	354	399	452	329	368	414	354	399	452
		k = 2	294	364	443	277	396	479	294	364	443	277	397	479	294	365	443	277	396	479
		k = 3	296	364	438	253	316	401	296	364	438	253	316	401	296	364	438	253	316	401
	Last donor	k = 1	09:30	09:53	10:18	09:36	09:54	10:17	9:30	9:53	10:18	9:37	9:54	10:17	9:30	9:55	11:33	9:36	9:54	10:16
		k = 2	11:28	11:54	12:19	11:37	11:59	12:21	11:28	11:54	12:19	11:37	11:59	12:21	11:28	11:55	12:19	11:37	11:58	12:21
		k = 3	13:34	13:51	14:17	13:24	13:48	14:14	13:34	13:51	14:17	13:24	13:48	14:14	13:34	13:51	14:17	13:24	13:48	14:14

Regarding donors, the most evident result is the reduction in the maximum cycle time in all configurations analysed. This reduction is more evident for unbooked donors in the configuration with prioritized queue management (*PRTY‐2‐B‐02*). At the same time, it is true that the minimum and the average cycle time are slightly increased for almost all configurations. However, the regularization of the cycle time with a reduction of the maximum peak is a desirable outcome for this metric. Compatible results were obtained for queue time.

Regarding resources, generally speaking, their saturation is slightly increased. However, the differences are minimal.

From the management’s viewpoint, the physicians’ used capacity in period k=1 was higher than without feedback. This proved that rescheduling was effective in better filling the first period. Coherently, the increment with respect with the baseline without feedback was lower in period k=2, and the physicians’ used capacity was even lower in k=3.

The results concerning the exit time of the last donor were similar to with those of physicians’ utilization. With respect to the case without feedback, the exit time was delayed in the first and second periods, where also physicians’ utilization was higher, while the exit time was lower in the third second period where physicians’ utilization was lower.

Overall, it is possible to conclude that the feedback loop to the BDAS achieved normalization of the results, for almost all KPIs considered, contributing to better management of the system. Further refinements could be achieved by iterating the feedback in a complete optimization‐simulation approach. In summary, the coupled use of the scheduler with the DES provides an inexpensive tool to manage a blood collection centre, enabling to combine mid‐term tactical decisions with short‐term operational ones, and verifying the feasibility of the former iteratively.

## Discussion

Even if the approach has been applied only to the case of AVIS Milan, the DES can be easily tailored to model other centres, due to its flexibility and generality, and the outcomes are expected to be confirmed also in these cases. In particular, the DES is built in a parametric form: this feature allows the testing of more scenarios than those included in this work, including (but not limiting to) donor arrival patterns and variations of shift durations. To extend the applicability, it would also be interesting to study how some parameters may evolve over time, such as no‐show and deferral rate, and to dynamically readapt the management of donors’ appointments in compliance.

Future research will extend the DES model. For example, we could identify other factors that impact the organization of blood donation centres, and fine‐tune the simulation parameters to other specific cases. At the same time, as in this work we only take into account whole blood donations, we will consider apheresis procedures to be incorporated into the model. Additionally, for regarding personnel, our DES model does not take into account differentiated work schedules and assumes that human resources are always available within the period, with no breaks. These two aspects could be integrated into the framework to better study human resource and staffing requirements for collection centres.

Finally, starting from the already implemented feedback from the simulator to the scheduler, we will extend the interaction to obtain a full optimization‐simulation scheme.

## Conclusions

In this paper, we study the operations in blood collection through a DES model developed to describe a general collection centre in a flexible way. The two goals are: to build a model that enables to study different configurations and work rules and to evaluate the effectiveness and feasibility of schedules defined at higher planning levels. A great effort has been made to make the model consistent with and adaptable to the characteristics of the centres, which is the main contribution of this work, based both on the interaction with AVIS Milan and on the analysis of works present in the literature.

Our DES model includes most of the key features of a collection centre, for example the simultaneous presence of booked and unbooked donors, and an assessment of the perspectives of all three stakeholders involved. With respect to the available literature, in which blood collection is not adequately addressed, we specifically address this step of the BDSC. We also underline that, thanks to the interaction with the BDAS, our DES model represents a crucial element for implementing the goals pursued by the scheduler in the real practice of providers.

Given the context of increasing demand and stationary budget faced by most blood centres, the proposed DES model and the decision framework in which it is included can provide an inexpensive aid for evidence‐based decision‐making. In this direction, the presented results prove that the tool can be effectively used in practice both to find out the best setting and to give feedback to a higher decision level (i.e. scheduler). In particular, the outcomes of the DES analysis can be beneficial for a series of activities such as centre dimensioning, daily operations improvement and staff shift management, either as a standalone tool or together with a higher level planning system.
